# Framework of behavioral indicators evaluating TB health promotion outcomes: a modified Delphi study of TB policymakers and health workers

**DOI:** 10.1186/s40249-015-0087-4

**Published:** 2015-12-15

**Authors:** Ying Li, John Ehiri, Daiyu Hu, Eyal Oren, Jia Cao

**Affiliations:** Department of Social Medicine and Health Service Management, Third Military Medical University, No.30 Gaotanyan Road, Shapingba district, Chongqing, China; Department of Health Promotion Sciences, Mel and Enid Zuckerman College of Public Health, University of Arizona, Tucson, USA; Chongqing Institute of TB Prevention and Treatment, Jiulongpo district, Chongqing, China; Department of Epidemiology & Biostatistics, Mel and Enid Zuckerman College of Public Health, University of Arizona, Tucson, USA; Institute of Toxicology, Third Military Medical University, No.30 Gaotanyan Road, Shapingba district, Chongqing, China

**Keywords:** Health promotion, Evaluation, Policymakers, TB healthcare workers, TB HCWs, Indicator, Delphi method

## Abstract

**Background:**

Although TB health promotion directed at policy makers and healthcare workers (HCWs) is considered important to tuberculosis (TB) control, no indicators currently assess the impact of such promotional activities. This article is the second in a series of papers that seek to establish a framework of behavioral indicators for outcome evaluation of TB health promotion, using the Delphi method. In the first article, we sought to establish a framework of behavioral indicators for outcome evaluation of TB health promotion among TB suspects and patients. The objective of this second article is to present an indicator framework that can be used to assess behavioral outcomes of TB health promotion directed at policy makers and HCWs.

**Methods:**

A two-round, modified Delphi method was used to establish the indicators. Sixteen experts who were knowledgeable and experienced in the field of TB control were consulted in Delphi surveys. A questionnaire was developed following 4 steps, and involved ranking indicators on a five-point Likert scale. The consensus level was 70 %. Median, mode, and Coefficient of variation (CV) were used to describe expert responses. An authority coefficient (Cr) was used to assess the degree of each expert’s authority.

**Results:**

Consensus was achieved following the two survey rounds and several iterations among the experts. For TB health-promotion activities directed at policymakers, the experts reached consensus on 2 domains (“Resource inputs” and “Policymaking and monitoring behaviors”), 4 subdomains (“Human resources” among others), and 13 indicators (“Human resources per 100,000 person” among others). For TB health-promotion activities directed at HCWs, the experts reached consensus on 5 domains (“Self-protective behaviors” among others), 6 sub-domains (“Preventing infection” among others), and 15 indicators (“Average hours of daily workplace disinfection by ultraviolet radiation” among others).

**Conclusions:**

This study identified a conceptual framework of core behavioral indicators to evaluate TB health-promotion activities directed at policymakers and HCWs involved in TB control. Validation in other parts of the world could lead to global consensus on behavioral indicators to evaluate TB health promotion targeted at policymakers and HCWs.

**Electronic supplementary material:**

The online version of this article (doi:10.1186/s40249-015-0087-4) contains supplementary material, which is available to authorized users.

## Multilingual abstracts

Please see Additional file 1 for translations of the abstract into the six official working languages of the United Nations.

## Background

Globally, the tuberculosis (TB) mortality rate has fallen by 41 % since 1990, and the world is on track to reach the global target of a 50 % reduction during 2015 [1]. However, global TB control still faces many challenges, with an estimated 8.7 million incident cases in 2011 and 1.4 million deaths from TB since 2011. Progress in responding to multidrug-resistant TB (MDR-TB) remains slow [1], particularly in high-burden countries where the incidence of MDR-TB is unacceptably high [2, 3]. In addition, global economic crises and reduced investments in health services threaten national tuberculosis control programs [1, 4, 5]. TB control constitutes a global public good with benefits shared by everyone [6]. Health policymakers and healthcare workers (HCWs) have important roles in efforts to control TB [7–10].

Given the population-wide benefits that are associated with investment in efforts to prevent the spread of TB, governments should play a leading role in TB control. Yet government neglect of TB control is one of the primary reasons for the worldwide persistence of the disease [7]. The political will and commitment of a country to fight TB are essential for effective disease control [7, 8]. Lessons from TB control in China demonstrate the importance of political commitment: TB has historically been endemic in China, and progress in TB control was slow during the 1990s and early part of 21^st^ century, mainly because of government neglect and limited healthcare resources [3, 5]. After 2003, however, China achieved great success in TB control due to significantly strengthened commitment and government leadership, increased funding, revised legislation related to TB case reporting, well-defined technical policies based on the Directly Observed Therapy-Short-Course (DOTS) strategy, and a modified free treatment policy [9]. Currently, however, global financial crises and economic recessions have led to decreased funding for TB control, not only in China but also in other countries [5]. Usually, in public health, it is through policy that technical expertise is translated into programs and interventions to promote the health and wellbeing of populations. Because policymakers work on behalf of governments to facilitate the development and implementation of programs and interventions, they play an important role in TB control. For these reasons, policymakers constitute a crucial target for TB health promotion, and it is crucial to identify a framework that can be used to evaluate the effectiveness of promotional activities targeted at them.

The performance of healthcare systems is closely related to the quantity, distribution, knowledge, skills, and motivation of their workforces, particularly those individuals delivering services [7]. Constraints on these human resources have been reported as one of the main barriers to TB control [10]. In 2003, national TB program (NTP) managers from 18 of the 22 TB high-burden countries ranked inadequate human resources (HR) as the first of the top five constraints against reaching global TB control targets set by the World Health Organization (WHO) [10]. The WHO *Global Plan to Stop TB 2006 – 2015* acknowledges that the main HR issues affecting tuberculosis control are insufficient quality, quantity, and distribution of HCWs [11]. Awareness is increasing that HRs must be addressed in order to reach millennium development goals (MDGs) [12]. In China, in addition to inadequate funding, a further difficulty in TB control is the shortage of trained HCWs. Many TB control facilities are staffed inadequately or by poorly trained and unmotivated HCWs [13]. Strong evidence indicates that a lack of qualified HCWs is associated with diagnostic delay in China [14–20]. Thus, training HCWs adequately in working with TB patients and in advocating for new programs and policies is an important strategy in TB-endemic low and middle income countries. Although some reports have been published on assessing the capacity of HCWs, these have only assessed the quantity of healthcare workers in TB-control institutions, including number, educational levels, and professional titles [21–24]. There still remain a paucity of evidence-based indicator frameworks for evaluating health promotion interventions that target HCWs involved in TB control.

Health promotion focuses not just on individual knowledge and behavioral changes, but also on policy changes and capacity building [25]. TB control needs multi-sector cooperation, including the concerted efforts of public health authorities, clinicians, policy makers, technical assistance agencies, laboratory specialists, and others [26]. TB health programs should educate healthcare providers (both public and private), community members, public health officials, and policymakers on TB prevention and control [27]. TB health-promotion programs should be comprehensive and include interventions for health service buyers (government/policymakers), health service providers (HCWs), health service users (TB suspects/patients), and the general public. To this end, in China, the 2008 *Guidelines on Enforcement of Chinese Tuberculosis Control Program* includes one chapter (Chapter 8) on comprehensive health promotion for health policymakers and HCWs in TB-control facilities, TB suspects and patients, contacts, students, migrants, and the public [28]. Undoubtedly, indicators to evaluate the outcomes of any health intervention program are important [29] and should be identified during the planning stage [30]. Although other major global diseases such as HIV/AIDS and non-communicable diseases have universally accepted and systematic indicator frameworks for assessing the outcomes of the health-promotion activities that address them [31, 32], global TB-control health promotion has no such indicators. In order to address this gap, our research group recently conducted a series of studies to identify frameworks of behavioral indicators to evaluate TB health-promotion outcomes. Evaluation indicators for each of the above target populations are complex and relatively independent. In our previous report, we sought to identify an indicator framework for assessing TB health-promotion activities targeted at TB suspects and TB patients [33]. In this paper, we seek to address a gap related to the lack of indicators for assessing TB health promotion targeted at policymakers and HCWs. Although our earlier study [33] and the current study followed similar Delphi methodology involving the same study population, data were collected separately for TB patients and suspects, and for TB policymakers and healthcare workers. TB patients and suspects are a unique population; thus, it is important to report separately the behavioral indicators of TB promotional activities directed at them in order to inform policy and practice with a more focused understanding and effective use of data. Given the traditional lack of emphasis on evaluating policy efforts, it also helps to report separately on the findings for the two target groups. Such an approach also helps to ensure that the value of the findings for policymakers is not lost in the findings for TB patients and suspects.

## Methods

This study was part of a larger project intended to explore a framework of behavioral indicators for evaluating TB health promotion targeted at various populations. We used a modified Delphi method described in a previous report [33] to establish an indicator framework to evaluate TB health promotion targeted at policymakers and HCWs. This method included two quantitative survey rounds that were completed from May to October 2012.

### Selection of Delphi experts

Selection of experts for the Delphi survey was described in detail in a previous report [33]. Here, we present a brief description of the selection process. Purposive sampling was used to select a panel of experts. Criteria included the following:

1. policymakers working in the field of TB control for at least 5 years;
2. TB HCWs with senior professional titles, significant knowledge of TB control, and extensive work experience in TB control in order to ensure expert authority; and
3. experts representing different geographic regions of China in terms of east, west, north, and south.

Sixteen experts were selected. Each received a phone call or an email requesting consent and describing the Delphi process and expectations regarding participation.

### Instrument

The instrument used for this modified Delphi method consisted of four parts, as reported previously [33]: instructions for completing the survey, a questionnaire containing indicators for ranking, information used to evaluate expert authority in TB control, and general information and background about the experts.

**Fig. 1:**
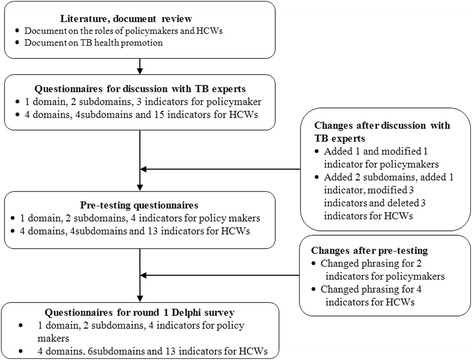
Flowchart of the Delphi survey questionnaire design. This figure describes the process in the development of the main Delphi survey questionnaire

Development of the Delphi survey instrument involved four steps (Fig. 1):

1. reviewing documents about TB health promotion and the roles of health-policymakers and HCWs [4–14, 21–24, 28, 34–43], and generating questionnaire items from the results for expert discussion;
2. organizing TB experts to discuss and modify the questionnaire items, and developing the draft Delphi survey questionnaire with domains, subdomains, and indicators for pre-testing;
3. pre-testing the Delphi questionnaire with a convenience sample of 3 TB HCWs; and
4. modifying the Delphi questionnaire according to preliminary analysis of pre-testing, and forming the final Delphi questionnaire.

This process resulted in a group of 1 domain, 2 subdomains, and 4 indicators for health policymakers, and a group of 4 domains, 6 subdomains, and 13 indicators for TB HCWs, together with the operational definitions and data-collection sources for all potentially relevant indicators (Table 1). The experts were then asked to assess the importance and feasibility of each indicator using a five-point Likert scale [33].

### Setting a consensus level

The Delphi method is based on panelists’ achieving consensus. However, experts can differ in interpretation and opinion, and it is typically difficult to gain 100 % agreement on all issues. In fact, no standard method or guidelines exist for determining appropriate consensus levels [44–51]. Therefore, the present study used a 70 % consensus level, as reported previously [33]:

- Consensus of inclusion: >70 % of participants scored the item ≥7;
- Consensus of exclusion: > 70 % subjects scored the item ≤5; and
- No consensus: item failed to meet either of the above criteria.

### Procedure

The procedure for administering the Delphi survey was reported previously [33]. Participants were asked to rank the importance and feasibility of the indicators in the first round. These rankings were then analyzed, and results were sent back to the participants for review and ratification. Items achieving consensus for exclusion were directly excluded from the questionnaire for the second-round survey; items suggested for modification were revised and kept in the second-round survey; additional items suggested as indicators were added to the second-round survey. Participants were asked to re-rank the consensus results from the first round in the second survey. Responses in the second round that reached 70 % consensus were determined to be appropriate for creating the indicator framework. Finally, the framework was presented to the experts, who participated in the concluding discussion during which the items achieving final consensus were selected for inclusion in the ultimate framework.

### Statistical analysis

Data analysis was undertaken by using the Statistical Package for the Social Sciences (SPSS) version 18.0. Median and mode were used to describe the central tendency of expert responses. Coefficient of variation (CV) was used to describe the variation in expert responses. The authority coefficient (Cr) was used to assess the degree of each expert’s authority, which was determined by judgment criteria for the indicator (Ca) and the expert’s familiarity with the indicators (Cs) [33].

### Ethical considerations

The study was funded by the National Natural Science Foundation of China (Grant # 81001297). The project proposal was approved by the Institutional Review Board of Third Military Medical University, Chongqing, China. All experts who participated in Delphi survey signed an informed consent form to confirm their voluntary participation.

## Results

### Characteristics of the experts

Two rounds of surveys were administered. During the first round, questionnaires were sent to seventeen experts, sixteen of whom responded. These sixteen content experts were from thirteen provinces/regions. All sixteen also completed the second-round survey. Descriptive information about the experts is available elsewhere [33].

### Authority of experts (Cr)

The expert authority coefficient ranged from 0.91 to 0.92 with an average Cr of 0.92, which indicated that the experts had a high degree of authority on the indicators under evaluation [33].

### Results of round-1 survey

The median score of the indicators’ importance and feasibility ranged from 7 to 9 for policymakers, except for a feasibility score of 5 on one indicator (“Number of policies TB control implemented”); modes ranged from 7 to 9; CVs were equal to, or less than 0.3, excepting modes of 0.4 and 0.5 for the feasibility of “Funds for TB control/person (RMB)” and “Number of policies TB control implemented,” respectively (Table 1). These results indicated that expert rankings showed a strong central tendency for all items in terms of importance and for most indicators in terms of feasibility, except for two indicators (“Funds for TB control” and “Number of policies TB control implemented”).

**Table 1:** Results of ratings in round 1 for health policymakers and TB HCWs

Item	Importance				Feasibility			
	Median	Mode	CV	Consensus (% score of >7)	Median	Mode	CV	Consensus (%score of >7)
Policymakers								
Domains								
Behaviors of policymaking	9	9	0.1	100	7	9	0.3	92.9
Subdomains								
Input for TB control	9	9	0.1	100	9	7	0.3	68.8
Policymaking	9	9	0.1	100	7	9	0.2	68.8
Indicators								
Funds for TB control	9	9	0.1	93.3	9	9	0.4	86.7
Human resources for TB control	9	9	0.1	100	9	9	0.3	71.4
Percentage of policies made for TB control	7	9	0.3	100	9	9	0.2	78.6
Number of TB-control policies implemented	9	7	0.3	80.4	5	9	0.5	92.9
TB HCWs								
Domains								
Self-protective behavior	9	9	0.1	93.3	9	9	0.2	93.4
Behaviors related to diagnosis of TB patients	9	9	0.1	100	7	7	0.3	73.3
Behaviors related to treatment of TB patients	9	9	0.1	100	7	7	0.2	86.7
Behaviors related to health education targeted at TB patients	9	9	0.1	100	7	7	0.2	80
Subdomains								
Breaking the chains of transmission (Prevention of infection)	9	9	0.1	100	7	9	0.3	93.3
Implementing clinic guidelines	9	9	0.1	100	7	7	0.3	66.7
Implementing standard treatment regimens	9	9	0	100	8.1	9	0.3	93.3
Visiting patients regularly	9	9	0.1	100	8.1	9	0.2	93.3
Content of health education	9	9	0.1	100	7	7	0.2	80
Time for health education	8.4	9	0.2	85.7	7	7	0.3	66.7
Indicators								
Hours/day of disinfecting workplace by ultraviolet radiation	9	9	0.2	93.4	9	9	0.1	100
Percentage of doctors ventilating workplace daily	9	9	0.2	85.7	9	9	0.3	78.6
Percentage of doctors wearing respirators	9	9	0.1	100	9	9	0.2	93.3
Handwashing after each patient appointment	7	9	0.4	66.7	9	9	0.3	86.7
Percentage of patients diagnosed according to clinic guidelines	9	9	0	100	7	7	0.3	66.7
Percentage of patients using a standard treatment regimen	9	9	0	100	8.1	9	0.1	100
Percentage of patients covered by DOT	9	9	0.2	92.8	9	9	0.1	100
Percentage of patients who received information on free treatment policy	9	9	0.2	83.3	9	9	0.1	93.3
Percentage of patients who received information on importance of adhering to treatment	9	9	0.1	100	9	9	0.2	86.7
Percentage of patients who received information on preventing TB transmission	9	9	0.1	100	9	9	0.3	73.3
Percentage of patients who received information on regular follow-up sputum microscopies	9	9	0.1	100	9	9	0.3	80
Percentage of patients who received information on drug side-effects	9	9	0.1	83.3	7.7	9	0.2	86.6
Average minutes of health education per patient	7	7	0.3	86.7	7	9	0.3	64.3

Note: UVGI refers to Ultraviolet germicidal irradiation

Both the medians and modes of the importance of two indicators for HCWs ranged from 7 to 9 among the experts; CVs were equal to, or less than 0.3, except for 0.4 for importance of one indicator (“Hand washing after appointment with each patient”) (Table 1). These results indicated that expert rankings showed a strong central tendency for all items in terms of feasibility and for almost all indicators in terms of importance, save for one indicator (“Hand washing after appointment with each patient”).

### Indicator screening after first-round survey

Based on the consensus criteria listed earlier, indicators with a consensus score of >7 by >70 % of the experts were considered appropriate, and indicators with a consensus score of ≤5 by >70 % of experts participants were excluded. After the round-one survey, consensus on inclusion was achieved as follows:

- *for policymakers*,
  - 1 domain (“Behaviors of policymakers”),
  - 3 indicators (“Funds for TB control,” “Human resources for TB control,” and “Percentage of policies made for TB control”);
- *for HCWs,*
  - 4 domains (“Self-protective behavior,” “Behaviors related to diagnosing TB patients,” “Behaviors related to treating TB patients” and “Behaviors related to health education targeted at TB patients”),
  - 5 subdomains (“Preventing infection,” “Implementing clinic guidelines,” “Implementing standard treatment regimens,” “Regular patient visits,” and “Content of health education”);
  - 11 indicators (“Hours/day of disinfecting workplace by ultraviolet radiation,” “Percentage of patients diagnosed according to clinic guidelines,” “Percentage of doctors ventilating workplace every day,” “Percentage of doctors wearing N-95 respirators,” “Percentage of patients using a standard treatment regimen,” “Percentage of patients covered by DOT,” “Percentage of patients receiving information on free treatment policy,” “Percentage of patients receiving information on importance of adherence to treatment,” “Percentage of patients receiving information on preventing transmission to others,” “Percentage of patients receiving information on regular follow-up sputum microscopy,” “Percentage of patients receiving information on side-effects of drugs”).

No indicators achieved consensus for exclusion (Table 1).

The results for policymakers indicated that all but one indicator (“Number of policies implemented”) in the first-round questionnaire were considered important and feasible. Experts also suggested that one indicator (“Number of policies implemented,” CV = 0.5 for feasibility) should be deleted, and one indicator (“Funds for TB control,” CV = 0.4 for feasibility), and one subdomain (“Input for TB control,” CV = 0.3 for feasibility) should be modified. Seven new items (1 domain, 1 subdomain, and 5 indicators) were recommended (Table 2). Ultimately, 2 domains, 4 subdomains, and 8 indicators were included for the second-round survey (Table 2).

**Table 2:** Results of rating in round 2 for health policymakers and TB HCWs

Item	Importance				Feasibility			
	Median	Mode	Consensus (% score of >7)	CV	Median	Mode	Consensus (% score of >7)	CV
Policymakers								
Domains								
Resource inputs	9	9	100	0.1	7	9	87.6	0.2
Behaviors of policymakers	9	9	100	0.1	7	9	87.6	0.2
Subdomains								
Financial resources	9	9	100	0	9	9	100	0.1
Human resources	9	9	100	0	9	9	100	0.1
Policymaking	9	9	100	0.1	9	9	93.8	0.2
Monitoring of policy implementation	9	9	100	0.1	7	9	80	0.2
Indicators								
(Full time/part time) human resources per 100,000 people)	9	9	100	0.1	9	9	100	0.1
Special funds for TB control/person	9	9	100	0	9	9	100	0.1
Funds for infrastructural construction in the past 5 years	9	9	87.6	0.2	9	9	81.3	0.2
Funds for infrastructural construction during the evaluation	9	9	87.5	0.2	9	9	81.3	0.2
Number of policies made for TB control	9	9	100	0.1	9	9	100	0.1
Satisfaction rate with policy (in terms of funding, decision making, implementing, monitoring)	9	9	100	0.1	7	7	75.3	0.2
Percentage of workplace meetings assigned to TB control	7	9	75.1	0.2	7	7	62.5	0.3
Percentage and number of policies monitored	8	9	93.8	0.2	7	9	75	0.2
TB HCWs								
Domains								
Self-protective behavior	9	9	100	0	9	9	100	0.1
Behaviors related to diagnosing TB patients	9	9	100	0.1	7	7	93.8	0.2
Behaviors related to tracing of TB suspects	9	9	100	0.1	9	9	93.8	0.2
Behaviors related to treating TB patients	9	9	100	0.1	8	9	93.8	0.2
Behaviors related to health education targeted at TB patients	9	9	100	0.1	7	7	81.3	0.2
Subdomains								
Breaking the chains of transmission (Prevention of infection)	9	9	100	0.1	8	9	87.5	0.2
Implementing clinic guidelines for TB patient diagnosis	9	9	93.3	0.1	7	7	80	0.2
Tracing TB suspects referred by non-TB control health facilities	9	9	93.8	0.2	9	9	86.7	0.2
Implementing standardized chemotherapy	9	9	93.8	0.1	9	9	100	0.1
Monitoring treatment	9	9	93.8	0.2	9	9	87.6	0.2
Content of health education	9	9	93.8	0.1	8	9	100	0.1
Time on health education	8	9	100	0.2	7	7	91.3	0.2
Indicators								
Hours of workplace UVGI (hours/day)	9	9	93.8	0.1	9	9	100	0.1
Percentage of doctors ventilating workplace everyday	9	9	100	0.1	9	9	100	0.1
Percentage of doctors wearing N-95 respirators	9	9	94.4	0.1	9	9	100	0.1
Percentage of patients diagnosed at implementing clinic	9	9	93.8	0.1	7	7	86.7	0.2
Percentage of TB suspects successfully traced	9	9	100	0.2	9	9	86.6	0.2
Percentage of patients who received standard treatment regimen	9	9	100	0.1	9	9	93.8	0.2
Percentage of patients covered by DOT	9	9	100	0.1	9	9	93.4	0.2
Percentage of patients who received information on free TB treatment policy	9	9	100	0.1	9	9	100	0.1
Percentage of patients who received information on importance of adherence to treatment	9	9	100	0.1	9	9	93.8	0.2
Percentage of patients who received information on regular follow-up sputum microscopies	9	9	100	0.1	9	9	100	0.1
Percentage of patients who received information on managing side-effects of anti-TB drugs	9	9	93.8	0.2	7	9	100	0.1
Percentage of patients who received information on a healthy lifestyle	9	9	93.8	0.2	7	9	87.6	0.2
Percentage of patients who received information on preventing TB transmission	9	9	93.8	0.2	7	9	87.6	0.2
Average minutes of health education per patient	8	9	87.5	0.2	7	7	93.8	0.2

Note: UVGI refers to Ultraviolet germicidal irradiation

As for the indicators for TB HCWs, experts suggested that one indicator (“Washing hands after appointment with each patient”) should be deleted from the second-round questionnaires, and 3 indicators should be modified. Experts also suggested that one domain (“Behaviors related to tracing TB suspects”), one subdomain (“Tracing TB suspects referred by non-TB control facilities”), and two new indicators (“Percentage of TB suspects successfully traced” and “Percentage of patients who received information on healthy lifestyle behaviors”) should be added (Table 2). Ultimately, 5 domains, 7 subdomains, and 14 indicators were included in the second-round survey questionnaire (Table 2).

### Results of round-two survey

Results of the indicators in the round-two survey showed that items in the questionnaires for both policymakers and TB HCWs were both important and feasible (median or mode for importance and feasibility for all indicators ranged from 7 to 9; CVs for all indicators were equal to, or less than 0.3) (Table 2). The results indicated that the expert rankings had a strong central tendency for most items in terms of importance and feasibility.

### Framework of behavioral indicators for assessing the impact of TB health promotion on health policymakers and TB experts

Based on our inclusion and exclusion criteria for the indicators for policymakers, consensus was achieved on the importance of all items and the feasibility of almost all items other than one indicator (“Percentage of meetings assigned to TB control in the workplace,” for which feasibility scored ≥7 by 62.5 % participants) (Table 2). No indicators achieved consensus for exclusion.

We organized a roundtable discussion with all the TB control experts to decide on final items for the framework. Following this discussion, “Percentage of meetings assigned to TB control in the workplace” was included in the framework, and 5 new indicators (“Percentage of bottom-up policymaking,” “Percentage of policies with measures to assess input, output, outcome, and impact,” “Percentage of policies with measures to promote cooperation of necessary sectors for policy implementation,” “Number of documents/policies for surveillance,” and “Percentage of evidence-based policymaking”) were added. Ultimately, 2 domains, 4 subdomains, and 13 indicators were included for policymakers (Table 3). For TB HCWs, inclusion consensus was achieved for all items in terms of both importance and feasibility based on consensus criteria (Table 2). Therefore, 5 domains, 7 subdomains, and 14 indicators were included for TB experts (Table 3).

**Table 3:** Conceptual framework for indicators for TB health promotion among Health policymakers and TB HCWs

Domain	Subdomain	Indicator	Measure	Data collection
Policymakers				
Resource inputs	Human resources	(Full time/part time) Human resources per 100,000 people	Number of full-time/part-time personnel (physicians, nurses) for TB control per 100 000 people (physicians/nurses/technicians per 100,000 people) and the quality of human resources	Review of documents and records in the department of human resources in the TB unit
	Financial resources	Special funds for TB control/person	Input of funds for TB control per person (RMB Yuan per person)	Review of documents in the financial department of the TB unit
		Funds for infrastructural construction in the past 5 years	Funds for infrastructural construction in the past 5 years (RMB Yuan)	Document and record review in TB-control health facilities
		Funds for infrastructural construction during the evaluation	Funds for infrastructural construction during the evaluation (RMB Yuan)	Document and record review in TB-control health facilities
Policymaking and monitoring behaviors	Policymaking	Percentage of meetings assigned to TB control in the workplace	Percentage of meetings assigned to TB control overall in work meetings, which evaluated attention of policymakers to TB control	Document and record review
		Percentage of policies made for TB control	Percentage of policies made for TB control (including policies for health insurance, free treatment, poverty alleviation, HCW incentives, activation of multi-sector participation in TB control, strategy, and guidelines)	Document review
		Rate of satisfaction with policy (in terms of funding, making, implementing, monitoring)	Rate of satisfaction with policy funding, policymaking, policy implementation, and policy monitoring	Policy implementation and beneficiary survey
		Percentage of policies made on the basis of evidence	Percentage of policies made based on evidence	Document and record review
		Percentage of policies made through a bottom-up approach	Percentage of policies made through a bottom-up approach	Document and record review
		Percentage of policies with measures to assess input, output, outcome, and impact	Percentage of policies that included measures to assess input, output, outcomes, and impact	Document and record review
		Percentage of policies with measures to promote cooperation of relevant sectors for policy implementation	Percentage of policies that included measures to promote cooperation of relevant sectors for policy implementation	Document and record review
	Surveillance of policy implementation	Percentage and number of policies monitored	Percentage and number of policies that were monitored and evaluated when implemented	Document and record review
		Number of documents/policies on Surveillance	Number of policy and strategy related to surveillance of related policy implementation	Document and record review
TB HCWs				
Self-protective behavior	Breaking the chains of transmission (Preventing infection)	Average hours spent daily on workplace disinfection by ultraviolet radiation everyday	Average hours spent on disinfecting the workplace by ultraviolet radiation each day in TB-control health facilities	HCW survey and observation study on HCWs
		Percentage of doctors who ventilate the workplace	Percentage of clinic doctors who ventilated the workplace every day in TB-control health facilities	HCW survey and observation study on HCWs
		Percentage of doctors who consistently wear N-95 respirators	Percentage of clinic doctors who wore N-95 respirators daily when working in TB-control health facilities	HCW survey and observation study on HCWs
Behaviors related to TB patient diagnosis	Implementing clinic guidelines in TB patient diagnosis	Percentage of patients diagnosed by implementing clinic guidelines	Percentage of patients diagnosed by implementing clinic guidelines in TB-control health facilities	TB patient survey and clinic records review
Behaviors related to tracing TB suspects	Tracing TB suspects referred by non-TB control health facilities	Percentage of TB suspects successfully traced	Percentage of TB suspects successfully referred to TB-control health facilities.	Clinic record review
Behaviors related to TB patient treatment	Implementing Standardized chemotherapy	Percentage of patients treated with standard treatment regimens	Percentage of patients who received standard treatment regimens recommended by WHO	TB patient survey and clinic record review
	Monitoring treatment	Percentage of patients covered by DOT	DOT refers to a standardized treatment regimen directly observed by an HCW or a community health worker for at least the first two months.	TB patient survey and clinic record review
Behaviors related to TB health education	Content of health education	Percentage of patients who received information on free treatment policy	Percentage of patients who received information about the TB free-treatment policy and who accurately understood this policy.	TB patient survey
		Percentage of patients who received information on importance of adhering to treatment	Percentage of patients receiving information on adherence to TB treatment and who accurately understood the importance of adhering to TB treatment.	TB patient survey
		Percentage of patients who received information on regular follow-up sputum microscopy	Percentage of patients receiving information about regular follow-up sputum microscopy and who accurately understood the importance of regular follow-up sputum microscopies.	TB patient survey
		Percentage of patients who received information on managing side-effects of anti-TB drugs	Percentage of patients who received information about managing side-effects of anti-TB drugs and who accurately understood the management of these side-effects.	TB patient survey
		Percentage of patients who received information on a healthy lifestyle	Percentage of patients who received information about a healthy lifestyle to complement TB treatment and who accurately understood how to maintain this healthy lifestyle	TB patient survey
		Percentage of patients who received information on preventing TB transmission	Percentage of patients who received information about preventing TB transmission and who accurately understood how to prevent TB transmission	TB patient survey
	Time on health education	Length of time spent on health education with each patient	Length of time spent on health education with each TB patient	TB patient survey, HCW observation study

## Discussion

Strong political commitment at various levels of government and significant resource development in health facilities in China have contributed to the success of the country’s TB program [3, 5, 9, 34]. The Chinese national *Guidelines on Enforcement of the Chinese Tuberculosis Control Program* (2008 version) emphasized health promotion targeted at both policymakers and HCWs [28]. After 5 years of guidelines implementation, it is important to develop an evaluation framework to assess outcomes [52]. Indicators are measures used to answer questions during the monitoring and evaluation of a health promotion intervention activity [32]. This study sought to establish a framework of behavioral indicators for assessing the impact of TB health promotion activities directed at policymakers and HCWs. It is hoped that the findings are relevant to national TB control programs, especially in the current harsh economic climate, in which programs must ensure higher cost-effectiveness.

Sufficient financial and human resources are critical for TB control, and appropriate financial mechanisms should be developed to ensure that TB control projects, especially in resource-poor settings, are well supported [14]. Political commitment and public policies on financial and human resource support at the national and local levels are among the primary support mechanisms for TB control [4–13]. Particularly for MDR-TB or extensively drug-resistant TB (XDR-TB) control, strong political commitment and adequate funding should underpin any strategy for addressing underlying societal and health-service determinants of MDR and XDR-TB [4]. Policymakers in government roles can influence resource availability and use for TB control, including financial and human resources [9].

Overall, governments in every region of China play critical roles in supplying resources and organizing activities related to TB control. Once the central Chinese government has made political and financial commitments, it tends to honor them. However, this may not be the case with many local governments, especially at the county level in economically disadvantaged areas [34]. The 2008 *Guidelines on Enforcement of Chinese Tuberculosis Control Program* stated that, in the domain of TB health promotion for policymakers, a primary objective is to improve their support of policy and finance [28]. Therefore, the framework to evaluate policymakers’ behavior should address behaviors related to allocating resources to TB control. The framework proposed in this study included one domain on resource input, two related subdomains (“Human” and “Financial resources”), one indicator for human resources, and three indicators related to financial inputs.

Health promotion specialists increasingly recognize that, to be effective in improving citizens’ health and quality of life, they must incorporate policy advocacy interventions as integral strategies [35]. Public policies at national and local levels are among the primary mechanisms for supporting TB control and can include financial support, human resource planning, health insurance, and even the reduction of TB stigma. In the past decade, TB control in China has benefited significantly from well-defined technical policies based on the DOTS strategy and from a modified free-treatment policy [9]. However, policymakers still need to focus more on creating appropriate policy environments for TB control. For example, in order to address financial challenges, new policies can be considered, such as integrating the national TB control program into health insurance schemes [15]. Other policy needs include using medical financing assistance for the poor to ensure access to TB control services for MDR-TB patients, using community health resources for primary health care in TB control [5], paying for TB care in public hospitals in order to discourage profiteering from service provision and illegal drug sales, and recognizing TB as an occupational disease to reduce job-related tuberculosis among HCWs [36]. Therefore, indicators in this area should cover policymaking behaviors. This study proposed a framework with one such domain (of policymaking and monitoring,) and two subdomains (“Policymaking and monitoring of policy implementation”).

This study proposed one indicator to evaluate the number of policies made for TB control. Furthermore, while the quality of each policy is equally important, it is difficult to assess quality in policymaking and the impacts of policy interventions [37]. We suggest satisfaction ratings as a way of assessing policy quality by both implementers and users. The question was posed as to whether an evidence-based or bottom-up approach employed in policymaking should be used to assess quality, because health research and policymaking in China usually operate in different environments without adequate communication [38]. Although different policies might have different goals and objectives, policies should be proposed and enacted with related strategies for assessing input, output, outcomes, and impact [37]. In addition, policy implementation involves multiple organizations, and within an organization, staff from different departments might be involved [39]. All these actors have to make their own choices as to how to implement their respective parts of a policy. Therefore, policies should include strategies for encouraging cooperation among multiple sectors (such as allocating all resources necessary to implement a policy) [37]. To this end, after final discussion with TB experts, four indicators (“Percentage of evidence-based policy making,” “Percentage of bottom-up policymaking,” “Percentage of policies with measures to assess input, output, outcome and impact,” and “Percentage of policies with measures to promote cooperation of necessary sectors for policy implementation”) were added to the framework for evaluating policymaking behaviors.

Monitoring is the continuous, systematic collection of data on specified indicators to provide indications of progress toward objectives and the achievement of intermediate results along the way. While effective monitoring is necessary for effective program management, it is not sufficient for assessing ultimate results [40]. Therefore, this study proposed two indicators (“Percentage and number of times policy implementation was monitored” and “Number of documents/policies for surveillance”) under the subdomain of “Monitoring policy implementation.”

Doctors, nurses, and laboratory staff in TB-control health facilities must be trained properly and kept updated on the latest policy and healthcare developments related to TB [41]. After training, it is important to evaluate the contribution of that training to improving both HCWs' productivity and the overall quality of tuberculosis control programs [13]. However, little data is available on human resources for TB control, particularly data on the quality of these resources. One survey on human resources for TB control among 19 high TB-burden countries (HBCs) in 2003 assessed staff numbers, skills (training courses and coverage of training), performance (the estimated time needed to treat a new smear sputum positive patient), and estimated HR gap [12]. This survey indicated that many HBC countries do not have accurate information on numbers, types, and distribution of staff involved in TB-control activities; staff attendance at training courses; or characteristics, duration, and intensity of training activities, particularly in China [12]. In China, available studies on the assessment capacity of HCWs [21–24] only included number, education levels, and professional titles. No studies have assessed actual skills related to TB control. The aims of TB health promotion for HCWs in the 2008 *Guidelines* included correct and timely diagnosis of TB patients, treatment of TB patients with a standard regimen, carrying out TB health education to TB suspects or patients, and precautionary practices to prevent infection among HCWs [28]. Thus, the 5 domains proposed in the indicator framework for HCWs (“Self-protective behaviors,” “Behaviors related to TB diagnosis,” “Behaviors related to tracing of TB patients,” “Behaviors related to TB treatment,” and “Behaviors related to health education”) are relevant and appropriate.

Measures effective in preventing HCWs from being infected with TB included updated periodic training to maintain awareness of potential risks and appropriate use of effective respiratory protection as well as active infection control procedures [42]. There are three levels of infection control measures within health care facilities: administrative (managerial), environmental, and personal [43]. Among those measures, ventilation, Ultraviolet germicidal irradiation (UVGI), and the use of respiratory protective equipment can be managed by HCWs. Thus, the framework proposed by this study included one subdomain (“Breaking the chains of transmission”) and 3 indicators.

### Strengths and limitations

This study was the first to develop a framework of behavioral indicators for TB health promotion among policymakers and HCWs. In addition, after two sound surveys, consensus for inclusion was achieved on all items except one (62.5 % consensus for the feasibility of “arrangement of TB control in the health worker”). Yet, the study has a number of limitations:

1. Additional experts for the Delphi survey could have been contacted for better representation. The study only included policymakers from TB control institutes, but did not include experts in other sectors whose activities influence policy and resources for TB control, including policymakers in the Bureau or Ministry of Finance, or policymakers in the Ministry of Civil Affairs. In addition, HCWs at the county level were not included on the expert panel. It is necessary to tailor the framework of indicators to HCWs at different levels of the health system (such as county/district and primary care) because HCWs at different levels have different responsibilities in relation to TB control [29].
2. The study focused on constructing indicators to evaluate behavioral changes of frontline clinic HCWs only. It is equally important to consider indicators for HCWs in different departments within TB-control health facilities (nurses, laboratory staff, and health promoters) because their responsibilities vary.
3. As a mostly qualitative approach, Delphi surveys typically provide a means of structuring group discussions, raising issues for debate, and identifying questions for further empirical inquiries that enhance reliability and validity [45]. Therefore this study organized roundtable discussions to decide on the final items included in the framework after the second-round survey. Though this study organized a “consensus conference” to discuss the validity of its Delphi results, the framework was not tested by a quasi-experimental design, a series of interviews, secondary documentary data collection, or focus-group discussions.
4. The title “framework” implies a comprehensive system or structure in TB care and control. One important item which our present study did not address, and which will benefit from future research attention, is the issue of TB-contact investigation.

## Conclusion and implications

Evaluating health promotion programs is challenging, as health promotion takes place within fluctuating and complex settings [29]. Consequently, implementation research should be carried out to tailor TB health-promotion frameworks to varying contexts. In addition, further studies are needed to construct indicators for evaluating TB health promotion at the individual level, including behavioral changes among HCWs in departments other than clinical settings (nurses, laboratory staff), and at environmental levels. The results of this study can be used as a basis for further research. However, before any performance indicator can be adopted, it needs to be defined clearly and tested for reliability, validity, and responsiveness (the ability to detect a significant change in performance). Therefore, more studies are desirable for testing the framework by a quasi-experimental design, a series of interviews, secondary documentary data collection, and focus-group discussions [53, 54].

## Additional file

Additional file 1:
**Multilingual abstracts in the six official working languages of the United Nations.** (PDF 296 kb)
