# Supplementary material for: Framework of behavioral indicators evaluating TB health promotion outcomes: a modified Delphi study of TB policymakers and health workers
Source: Infect Dis Poverty. 2015 Dec 15;4:56. doi: 10.1186/s40249-015-0087-4 (PMC4678709; doi:10.1186/s40249-015-0087-4)
Supplement: Additional file 1: — Multilingual abstracts in the six official working languages of the United Nations. (PDF 296 kb) [file 40249_2015_87_MOESM1_ESM.pdf]

Translation of the abstract into the six official working languages of the United Nations

إطار عمل للمؤشرات السلوكية المستخدمة في تقييم نتائج أنشطة تعزيز الرعاية الصحية لحالات السل: دراسة بطريقة دلفي معدَّلة وموجَّهة لصنّاع السياسات والعاملين في الرعاية الصحية لحالات السل

ينج لي، جون إيهيري, دايو هو، إيال أورين، جيا تشاو

الملخص

الخلفية: بالرغم من أن أنشطة تعزيز الرعاية الصحية لحالات السل (TB)، والمقدَّمة إلى صنّاع السياسات والعاملين في مجال الرعاية الصحية (HCW)، عامل مهم في مكافحة مرض السل، لا توجد مؤشرات حالية لتقييم تأثير مثل هذه الأنشطة. وهذا المقال هو الثاني في سلسلة من الأوراق البحثية التي تسعى إلى إنشاء إطار عمل للمؤشرات السلوكية لتقييم نتائج أنشطة تعزيز الرعاية الصحية لمرضى السل، باستخدام طريقة دلفي (Delphi). في المقال الأول، سعينا إلى إنشاء إطار عمل للمؤشرات السلوكية الهادفة إلى تقييم نتائج أنشطة تعزيز الرعاية الصحية لحالات السل بين الأفراد المشتبه في إصابتهم بالمرض والمرضى الفعليين. لكن الهدف من هذا المقال الثاني هو تقديم إطار عمل للمؤشرات يمكن استخدامه في تقييم النتائج السلوكية لأنشطة تعزيز الرعاية الصحية لحالات السل، وهي الأنشطة المقدَّمة لصنّاع السياسات والعاملين في مجال الرعاية الصحية.

الطرق: تم استخدام طريقة دلفي معدَّلة من جولتين لإعداد المؤشرات. وقد جرت استشارة 16 خبيرًا ممن يملكون معرفة وخبرة واسعة في مجال مكافحة السل، في استبيانات دلفي. وتم وضع استبيان باتباع 4 خطوات، واشتمل على تصنيف للمؤشرات على مقياس "ليكرت" مكوَّن من 5 نقاط. وكان مستوى التوافق هو 70%. وقد استُخدم الوسيط، والمنول، ومعامل الاختلاف (CV) لوصف إجابات الخبراء. بينما استُخدم معامل القدرة المعرفية (Cr) لتقييم درجة القدرة المعرفية لكل خبير.

النتائج: جرى التوصل إلى توافق في الآراء بعد جولتي الاستبيان والعديد من دورات التكرار بين الخبراء. بالنسبة إلى أنشطة تعزيز الرعاية الصحية المقدَّمة إلى صنّاع السياسات، توصل الخبراء إلى توافق في مجالين اثنين ("مدخلات الموارد" و"سلوكيات وضع السياسات والمراقبة")، و4 مجالات فرعية (من بينها "الموارد البشرية")، و13 مؤشرًا (من بينها "الموارد البشرية لكل 100000 شخص"). أما بالنسبة إلى أنشطة تعزيز الرعاية الصحية المقدَّمة إلى العاملين في مجال الرعاية الصحية، توصل الخبراء إلى توافق في 5 مجالات (من بينها "سلوكيات الحماية الذاتية")، و6 مجالات فرعية (من بينها "الوقاية من العدوى")، و15 مؤشرًا (من بينها "متوسط ساعات تطهير مكان العمل بالأشعة فوق البنفسجية يوميًا").

الخلاصة: حددت هذه الدراسة إطار عمل مفاهيميًا للمؤشرات السلوكية الأساسية الهادفة إلى تقييم أنشطة تعزيز الرعاية الصحية لحالات السل، وهي الأنشطة المقدَّمة لصنّاع السياسات والعاملين في مجال الرعاية الصحية ممن يشاركون في مكافحة مرض السل. ويمكن أن يؤدي التصديق على صلاحية الإطار في أماكن أخرى من العالم إلى التوصل إلى توافق عالمي حول المؤشرات السلوكية المستخدمة في تقييم أنشطة تعزيز الرعاية الصحية لحالات السل، وهي الأنشطة المقدَّمة إلى صنّاع السياسات والعاملين في مجال الرعاية الصحية.

Translated from English version into Arabic by Ahmed Samir Yahia, through

TRANSLATORS WITHOUT BORDERS

决策者和医务人员结核病健康促进的行为评价指标体系框架：Delphi 法

李颖，John Ehiri，胡代玉，Eyal Oren，曹佳

摘要

引言：虽然对决策者和医务人员进行结核病健康促进是结核病控制健康促进非常重要的内容，但是一直没有相应的对其行为改变效果进行评价的指标体系。该报道是采用 Delphi 法构建结核病健康促进行为评价指标体系构建系列研究中的第二篇。前一篇报道针对结核病可疑患者和患者的结核病健康促进的行为改变效果进行评价的指标体系构建。这篇报道主要目的是构建针对决策者和医务人员结核病健康促进后行为改变

效果进行评价的指标体系构建。
方法：该研究使用了两轮修改 Delphi 专家咨询法进行指标体系构建。我们对 16 位结核病防治专家进行了咨询。五点李克氏专家咨询表用于专家咨询，该咨询表是通过 4 步研制而成。一致率设定为 70%。用中位数、众数和协调系数等统计指标描述专家咨询的结果，权威度用于评价所选专家的权威性。
结果：通过两轮的咨询和几次会议讨论，专家的意见达成一致。针对决策者，形成了两个一级指标（资源投入和政策制定和监测）、4 个二级指标（人力资源等）和 13 个三级指标（每万人口的结核病人力资源等）。针对医务人员的指标体系包括 5 个一级指标（自我保护行为等）、6 个二级指标体系（预防感染等）和 15 个三级指标（工作场所平均每天紫外线消毒的时间）
结论：该研究提出了针对决策者和医务人员结核病健康促进行为改变效果的评价指标体系框架。该指标体系的提出为世界上其他地区决策者和医务人员结核病健康促进的效果评价提供重要参考。

Translated from English version into Arabic by Li Ying

Cadre d’indicateurs comportementaux pour l’évaluation des résultats de la promotion des mesures antituberculeuses : étude Delphi modifiée des décideurs et professionnels de la santé engagés contre la tuberculose

Ying Li, John Ehiri, Daiyu Hu, Eyal Oren, Jia Cao

Résumé

Contexte : Bien que l’importance de la promotion sanitaire antituberculeuse à l’adresse des décideurs politiques et des professionnels de la santé soit reconnue dans la lutte contre la tuberculose, il n’existe pas actuellement d’indicateurs évaluant l’impact de ces activités. Notre article est le deuxième d’une série visant à établir un cadre d’indicateurs comportementaux pour évaluer les résultats de la promotion sanitaire antituberculeuse en utilisant la méthode Delphi. Dans le premier article, nous avons tenté de formuler ces indicateurs dans le cadre de la promotion sanitaire auprès des patients et des cas suspects de tuberculose. L’objectif de ce deuxième article est de présenter un cadre d’indicateurs pouvant être utilisé pour évaluer les résultats comportementaux de la promotion sanitaire antituberculeuse à destination des décideurs politiques et des professionnels de la santé.
Méthodes : Une méthode Delphi modifiée en deux tours a été utilisée pour identifier les indicateurs. Seize experts bien informés et expérimentés en matière de lutte contre la tuberculose ont été consultés dans le cadre d’enquêtes suivant cette méthode. Un questionnaire en 4 volets a été développé, avec des indicateurs de classement sur une échelle de Likert à cinq points. Le niveau de consensus était de 70 %. La médiane, le mode et le coefficient de variation (CV) ont été utilisés pour décrire les réponses des experts. Un coefficient d’autorité a été utilisé pour évaluer le degré d’autorité de chaque experts.
Résultats : Le consensus a été atteint après les deux tours d’enquête et plusieurs itérations parmi les experts. Sur les activités de promotion sanitaire antituberculeuse destinées aux décideurs politiques, les experts sont parvenus au consensus dans 2 domaines (« intrants » et « comportements de décision politique et de surveillance »), 4 sous-domaines (entre autres « ressources humaines ») et 13 indicateurs (entre autres « ressources humaines par 100 000 personnes »). Pour la promotion auprès des professionnels de la santé, les experts sont parvenus au consensus dans 5 domaines (entre autres « comportements d’autoprotection »), 6 sous-domaines (entre autres « prévention de l’infection ») et 15 indicateurs (entre autres « temps moyen de désinfection quotidienne des postes de travail par rayonnement ultraviolet »).
Conclusions : L’étude a identifié un cadre conceptuel d’indicateurs comportementaux centraux permettant

l’évaluation des activités de promotion sanitaire antituberculeuse adressées aux décideurs politiques et aux professionnels de la santé engagés dans la lutte contre la tuberculose. La validation de ce cadre dans d’autres parties du monde permettrait de parvenir à un consensus sur les indicateurs nécessaires à cette évaluation.

Translated from English version into French by Suzanne Assenat, through

TRANSLATORS WITHOUT BORDERS

Система поведенческих показателей для оценки результатов пропаганды профилактики заболевания туберкулезом: модифицированное прогнозирование по дельфийскому методу, реализованное разработчиками стратегии и специалистами в сфере здравоохранения

Йинг Ли, Джон Эхири, Дайю Ху, Эял Орен, Джиа Као

Краткое описание

Базовая информация: Хотя пропаганда профилактики заболевания туберкулезом среди разработчиков стратегии и специалистов в сфере здравоохранения считается важным для обеспечения контроля распространения туберкулеза, на сегодняшний день не существует объективных показателей, которые бы оценивали воздействие таких пропагандистских мероприятий. Настоящая статья является второй в серии документов, направленных на создание системы поведенческих показателей для оценки результатов пропаганды профилактики туберкулеза с использованием прогнозирования по дельфийскому методу. В первой статье мы пытались создать систему поведенческих показателей для оценки результатов пропаганды профилактики туберкулеза среди больных туберкулезом и лиц, входящих в группу риска по данному заболеванию. Цель второй статьи состоит в том, чтобы представить систему показателей, которая может быть использована для оценки поведенческих результатов пропаганды профилактики туберкулеза среди разработчиков стратегии с специалистов в сфере здравоохранения.

Методика: Для определения показателей использовалось двухфазное, модифицированное прогнозирование по дельфийскому методу. Шестнадцать экспертов, которые обладали определенными знаниями и опытом в сфере контроля распространения туберкулеза, выполняли роль консультантов в исследованиях по дельфийскому методу. Была разработана анкета из 4 частей, которая включает в себя показатели оценки по шкале Лайкерта. Удалось достигнуть 70-процентного консенсуса. Для описания ответов экспертов использовались срединные значения, наиболее вероятные значения и коэффициенты вариации. Коэффициент авторитетности был использован для оценки степени авторитетности мнения каждого из экспертов.

Результаты: После двух этапов исследования и обсуждения экспертами был достигнут консенсус. В отношении мероприятий по пропаганде профилактики туберкулеза среди разработчиков стратегии эксперты достигли консенсуса в двух областях («Обеспечение ресурсов» и «Разработка стратегии и мониторинг поведения»), 4 подобластях («Управление персоналом» и др.) и по 13 показателям («Количество персонала на 100000 человек» и др.). В отношении мероприятий по пропаганде профилактики туберкулеза среди специалистов в сфере здравоохранения эксперты достигли консенсуса в 5 областях («Поведение, направленное на самозащиту» и др.), 6 подобластях («Профилактика

инфицирования» и др.) и по 15 показателям («Среднее количество часов ежедневной дезинфекции рабочего места с помощью ультрафиолетовой радиации» и др.).
Выводы: Настоящее исследование позволило выявить концептуальную систему ключевых поведенческих показателей для оценки мероприятий по пропаганде профилактики туберкулеза среди разработчиков стратегии и специалистов в сфере здравоохранения, отвечающих за контроль распространения туберкулеза. Реализация результатов исследования в других частях мир может привести к глобальному консенсусу по поведенческим показателям для оценки пропаганды профилактики туберкулеза среди разработчиков стратегии и специалистов в сфере здравоохранения.

Translated from English version into Russian by Irina Zayonchkovskaya, through

Marco de indicadores de comportamiento que evalúan los resultados de la promoción de la salud para la TB Un estudio Delphi modificado de legisladores y trabajadores de la salud en lo que respecta a la TB

Ying Li, John Ehiri, Daiyu Hu, Eyal Oren, Jia Cao

Resumen
Antecedentes: Si bien se considera que la promoción de la salud para la TB dirigida a legisladores y trabajadores de la salud es importante para el control de la tuberculosis (TB), no existen en la actualidad indicadores que evalúen el impacto de dichas actividades de promoción. Este artículo es el segundo en una serie de artículos que tienen como objetivo establecer un marco de indicadores de comportamiento para la evaluación de resultados de la promoción de la salud para la TB mediante el uso del método Delphi. En el primer artículo buscamos establecer un marco de indicadores de comportamiento para la evaluación del resultado de la promoción de salud para la TB en pacientes con TB e individuos con sospecha de TB. El objetivo de este segundo artículo es presentar un marco indicador que pueda ser utilizado para evaluar los resultados de comportamiento de la promoción de la salud para la TB dirigida a legisladores y trabajadores de la salud.
Métodos: Para establecer los indicadores se utilizó un método Delphi modificado en dos rondas. Mediante encuestas Delphi se consultó a dieciséis expertos con conocimiento y experiencia en el campo del control de la TB. Se desarrolló un cuestionario que seguía 4 pasos, y que solicitaba que se clasificaran los indicadores en una escala Likert de cinco puntos. El nivel de consenso fue del 70%. Se utilizaron media, moda y coeficiente de variación para describir las respuestas de los expertos. Se utilizó un coeficiente de autoridad para evaluar el grado de autoridad de cada experto.
Resultados: Se logró el consenso luego de dos rondas de encuestas y varias iteraciones entre los expertos. Para las actividades de promoción de la salud para la TB dirigidas a legisladores, los expertos lograron el consenso en 2 campos (”Ingreso de recursos” y “Comportamientos de diseño de políticas y monitoreo”), 4 sub-campos (”Recursos Humanos” entre otros), y 13 indicadores (”Recursos humanos por cada 100,000 personas” entre otros). Para las actividades de promoción de la salud para la TB dirigidas a los trabajadores de la salud, los expertos lograron el consenso en 5 campos (”Comportamientos de auto-protección” entre otros), 6 sub-campos (”Prevención de la infección”, entre otros), y 15 indicadores (”Horas promedio por día de desinfección por

radiación ultravioleta en el lugar de trabajo” entre otros).

Conclusiones: Este estudio identificó un marco conceptual de indicadores claves de comportamiento para evaluar las actividades de promoción de la salud para la TB dirigidas a legisladores y trabajadores de la salud involucrados en el control de la TB. La validación en otros lugares del mundo podrá llevar a un consenso global sobre los indicadores de comportamiento para evaluar la promoción de la salud para la TB dirigida a legisladores y trabajadores de la salud.

Translated from English version into Spanish by Maria Alejandra Aguada, through

TRANSLATORS
WITHOUT BORDERS
